# SOCIAL SERVICE DIRECTOR PRIORITIES IN RESOLVING CONFLICTS IN NURSING HOMES

**DOI:** 10.1093/geroni/igad104.0107

**Published:** 2023-12-21

**Authors:** Nancy Kusmaul, Amy Roberts, Mercedes Bern-Klug

**Affiliations:** University of Maryland Baltimore County, Baltimore, Maryland, United States; Miami University, Oxford, Ohio, United States; University of Iowa, Iowa City, Iowa, United States

## Abstract

Social workers in nursing homes serve many critical roles such as assessing for residents’ unmet psychosocial needs, providing support to residents and families at end of life, and resolving conflicts between residents, families, and staff. In their roles, they often need to balance conflicting priorities and determine whose rights or needs should take priority. Little is known about how these conflicts affect the social workers in their jobs. This presentation will report the results of a content analysis of the answers to two open ended questions, “What do you like about your job?” and “What would you change about your job?” in a nationally representative sample of nursing home social service directors. The results illustrate directors’ views of what they value in terms of helping residents, families, and staff navigate change, difficult conversations, and conflicting interests. Social service directors report that their priorities are residents and their families, specifically advocating for resident needs. They also report needing to advocate for the role of social work on the interdisciplinary team. They report that family dynamics are messy, but do not discuss how they prioritize within and between residents, their family members, and nursing home staff. More research is needed to understand the decision making process that social workers employ in decision-making about priorities.

